# Supplementary material for: Patterns of Intron Gain and Loss in Fungi
Source: PLoS Biol. 2004 Nov 30;2(12):e422. doi: 10.1371/journal.pbio.0020422 (PMC532390; doi:10.1371/journal.pbio.0020422)
Supplement: Table S1 — Also available at http://genes.mit.edu/NielsenEtAl/. (4.3 MB ZIP). [file pbio.0020422.st001.zip › NielsenEtAl/html/608.html]

AN6330.1.NCU07700.1.MG01742.1.FG09574.1


```
 CLUSTAL W (1.82) Multiple Sequence Alignments - Introns Inserted


Sequence 1: MG01742.1	869 aa
Sequence 2: FG09574.1	832 aa
Sequence 3: NCU07700.1	844 aa
Sequence 4: AN6330.1	844 aa
Alignment Length: 877 aa
Number Identitical Residues: 622 aa
Alignment Score (without introns) 26329


MG01742.1 	---~----M~VK2ALMDKPCNVRNMSVIAHV~DHGKSTLTDSLLAKAGIISTAKAGDQRA
NCU07700.1	MHS~FTIDE~IR~ALMDKPTNVRNMSVIAHV~DHGKSTLTDSLLAKAGIISSGKAGEARA
FG09574.1 	---~-----~--~--MDKPTNVRNMSVIAHV~DHGKSTLTDSLLAKAGIISTAKAGDARA
AN6330.1  	MVN2FTIEE0LR~SLMDRKANIRNMSVIAHV1DHGKSTLSDSLVSRAGIIAGAKAGDARF
          	  .  : .     : **:  *:********* *******:***:::****: .***: * 

MG01742.1 	TDTRADEQERGITIKSTAISLYGNLPSDDDLKDIVGQKVDG~KDFLINLIDSPGHVDFSS
NCU07700.1	TDTRADEQERGITIKSTAISLYGTLPDEEDIKDIVGQKTDG1KDFLINLIDSPGHVDFSS
FG09574.1 	TDTRADEQERGITIKSTAISLYGQLGEDDDVADIVGQKTDG~KDFLINLIDSPGHVDFSS
AN6330.1  	MDTRPDEQERGITIKSTAISLYAKFADEEDIKEIP-QAVDG~NEFLINLIDSPGHVDFSS
          	 ***.*****************. : .::*: :*  * .** ::****************

MG01742.1 	EVTAALRVTDGALVVVDTVEGVCVQTETVLRQALGERIKPVIIINKVDRALLELQVTKED
NCU07700.1	EVTAALRVTDGALVVVDTVEGVCVQTETVLRQALGERIKPVVVINKVDRALLELQVSKED
FG09574.1 	EVTAALRVTDGALVVVDTVEGVCVQTETVLRQALGERIKPVIIINKVDRALLELQVSKED
AN6330.1  	EVTAALRVTDGALVVVDCVSGVCVQTETVLRQALTERIKPVLIINKVDRSLLELQVEKED
          	***************** *.************** ******::******:****** ***

MG01742.1 	LYQSFSRTIESVNVIISTYFDKSLGDVQVYPYKGTVAFGSGLHGWAFTVRQFAVRYAKKF
NCU07700.1	LYQSFSRTIESVNVIISTYFDKSLGDVQVYPDRGTVAFGSGLHGWAFTIRQFATRYAKKF
FG09574.1 	LYQSFSRTIESVNVIISTYLDKSIGDIQVYPDKGTVAFGSGLHGWAFTVRQFAVRYAKKF
AN6330.1  	LYQSFLRTVESVNVIIATYEDKALGNVQVYPEKGTVAFGSGLHGWAFTVRQFAVKFAKKF
          	***** **:*******:** **::*::**** :***************:****.::****

MG01742.1 	GVDRNKMMERLW0GDNYFNPATKKWTTKS-EHEGKQLERAFNQFILDPIFRIFKAVMNFK
NCU07700.1	GVDRNKMMERLW~GDNYFNPKTKKWTKNG-TYEGKELERAFNQFILDPIFKIFSAVMNFK
FG09574.1 	GVDKNKMMERLW~GDNYFNPKTKKWTKNG-TYEGKQLERAFNQFILDPIFKIFSAVMNFK
AN6330.1  	GVDRKKMLERLW~GDNYFNPKTKKWTKTQPEVDGKPVERAFNMFILDPIYKIFQLVTNDK
          	***::**:**** ******* *****.. .  :** :***** ******::**. * * *

MG01742.1 	RDEVDQLLAKLELKLPTEDKEKEGKQLLKAVMRTFLPAADSLLEMMILHLPSPVTAQRYR
NCU07700.1	KDEVAALLEKLNLKLATDDREKEGKQLLKAVMKAFLPAADCLLEMMILHLPSPVTAQAYR
FG09574.1 	KEEITTLLEKLNLKLNAEDREKEGKQLLKAVMRTFLPAADSLLEMMILHLPSPATAQKYR
AN6330.1  	KDQIPALLEKIEVKLANDEKDLTGKQLLKTIMRKFLPAADAMLEMICIHLPSPVTAQKYR
          	::::  ** *:::**  ::::  ******::*: ******.:***: :*****.*** **

MG01742.1 	VETLYEGPPDDAAAIAIRDCDPKGPLMLYVSKMVPTSDKGRFYAFGRVFAGTVRSGLKVR
NCU07700.1	AETLYEGPQDDEAAMAIKTCDPKGPLMLYVSKMVPTSDKGRFYAFGRVFAGTVRSGLKVR
FG09574.1 	AETLYEGPIDDEAAIGIRDCDPKGPLMLYVSKMVPTSDKGRFYAFGRVFSGTVRSGLKVR
AN6330.1  	AETLYEGPQDDEAFAGIRDCDPKAPLMLYVSKMVPTSDKGRFYAFGRVYAGTVKSGLKVR
          	.******* ** *  .*: ****.************************::***:******

MG01742.1 	IQGPNYTPGKKEDLFIKAVQRTVLMMGGKVEPIDDMPAGNIVGLVGIDQFLLKSGTLTTD
NCU07700.1	IQGPNYTPGKKEDLFIKAIQRTVLMMGGKVEPIDDMPAGNIVGLVGIDQFLLKSGTLTTS
FG09574.1 	IQGPNYVPGKKEDLFIKAIQRTVLMMGGKVEPIDDMPAGNIVGLVGIDQFLLKSGTLTTS
AN6330.1  	IQGPNYTPGKKDDLFIKAIQRTILMMGRFVEPIEDVPAGNIVGLVGVDQFLLKSGTLTTS
          	******.****:******:***:****  ****:*:**********:************.

MG01742.1 	ETAHNLKVMKFSVSPVVQQSVQVKNAQDLPKLVEGLKRLSKSDPCVLTMTNESGEHIVAG
NCU07700.1	ETAHNMKVMKFSVSPVVQRSVQVKNAQDLPKLVEGLKRLSKSDPCVLTFSNESGEHVVAG
FG09574.1 	ETAHNLKVMKFSVSPVVQRSVQVKNAQDLPKLVEGLKRLSKSDPCVLTMTSESGEHVVAG
AN6330.1  	ETAHNLKVMKFSVSPVVQRSVEVKNAGDLPKLVEGLKRLSKSDPCVLTMINESGEHVVAG
          	*****:************:**:**** *********************: .*****:***

MG01742.1 	AGELHLEICLKDLEEDHAGVPLIISDPVVQYRETVAGKSSMTALSKSPNKHNRLYMNAEP
NCU07700.1	AGELHLEICLNDLENDHAGVPLTISDPVVQYRETVAGKSSMTALSKSPNKHNRLYMVAEP
FG09574.1 	AGELHLEICLKDLEEDHAGVPLIISDPVVQYRETVTGKSSITALSKSPNKHNRLYMVAEP
AN6330.1  	AGELHLEICLKDLEEDHAGVPLRISDPVVSYRETVSGTSSMTALSKSPNKHNRLYLTAEP
          	**********:***:******* ******.*****:*.**:**************: ***

MG01742.1 	LTEELAQLIDDGKITPRDDFKARARVLADEHGWDVTDARKIWTFGPDTNGPNLLVDQTKA
NCU07700.1	LEEDLCLAIEAGKITPRDDFKARARILADDFGWDVTDARKIWAFGPDTNGANLLVDQTKA
FG09574.1 	IDEELSLAIEGGKVSARDDFKARARVLADDFGWDVTDARKIWTFGPDGTGANLLVDQTKA
AN6330.1  	LDEEVSKAIEEGKINPRDDFKARARILADEYNWDVTDARKIWCFGPDTTGANLLVDQTKA
          	: *::.  *: **:..*********:***:..********** **** .*.*********

MG01742.1 	VQYLNEIKDSVVSGFQWATREGVLAEEPMRGIRFNILDVTLHADAIHRGAGQLMPTTRRV
NCU07700.1	VQYLNEIKDSVVSGFQWATREGPIGEEPMRSIRFNILDVTLHADAIHRGGGQIIPTARRV
FG09574.1 	VQYLNEIKDSVVSGFQWATREGPVAEEPMRSCRFNILDVTLHADAIHRGGGQIIPTARRV
AN6330.1  	VQYLNEIKDSVVSGFQWATREGPVAEEPMRSIRFNILDVTLHADAIHRGGGQIIPTARRV
          	********************** :.*****. *****************.**::**:***

MG01742.1 	LYASTLLAEPAILEPVFLVEIQVPEQAMGGVYSVLTRRRGMVFNEEQRPGTPLFTIKAYL
NCU07700.1	LYAATLLAEPSLLEPVFLVEIQVPEQAMGGVYGVLTRRRGHVFGEEQRPGTPLFTIKAYL
FG09574.1 	LYAASLLAEPALLEPVYLVEIQVPEQAMGGVYGVLTRRRGHVFSEEQRPGTPLFNIKAYL
AN6330.1  	LYAATLLAEPGILEPIFNVEIQVPEQAMGGIYGVLTRRRGHVYTEEQRVGTPLFTVKAYL
          	***::*****.:***:: ************:*.******* *: **** *****.:****

MG01742.1 	PVMESFGFNADLRQGTSGQAFPQSVFDHWQVLQGGSPLDATSKTGTVVQNTRKRKGLKPE
NCU07700.1	PVMESFGFNGDLRAATSGQAFPQSVFDHWERLPGGSPLDSTSKVGQIVQEMRKRKGLKVE
FG09574.1 	PILESFGFNGDLRQATSGQAFPQSVFDHWQILPGGSPLDATSKVGAIVTTMRKRKGVKEI
AN6330.1  	PVNESFGFPGELRQATGGQAFPQSVFDHWAVLPGGSPLDPTTKPGQIVAEMRKRKGIKEQ
          	*: ***** .:** .*.************  * ******.*:* * :*   *****:*  

MG01742.1 	VPGVEN0NHQYSTTTSCKRLLAKLVYPILDPFSSITQQGRLHWQ
NCU07700.1	VPGYEN0------------YYDKL--------------------
FG09574.1 	VPGVEN0------------YYDKL--------------------
AN6330.1  	VPGYEN0------------YYDKL--------------------
          	*** **                **
```
